# Neuromonitoring of patients with severe traumatic brain injury at the bedside

**DOI:** 10.1186/cc14531

**Published:** 2015-03-16

**Authors:** M Aries, JG Regtien, M Czosnyka, J Donnelly, P Smielewski

**Affiliations:** 1 UMCG, Groningen, the Netherlands; 2 Addenbrookes Hospital, University of Cambridge, Cambridge, UK

## Introduction

Measurement of intracranial pressure (ICP) and arterial blood pressure is used to derive cerebral perfusion pressure (CPP) and to guide targeted therapy of severe traumatic brain injury (TBI) necessitating ICU admission. In this review we discuss the evidence for ICP monitoring, CPP calculation, and ICP/CPP-guided therapy after severe TBI. Despite its widespread use, there is currently no class I evidence that ICP/CPP-guided therapy improves outcomes. Similarly, no class I evidence can currently advise the ideal CPP.

## Methods

A review of current literature with special focus on autoregulation (PRx)-guided CPP treatment in TBI patients.

## Results

Optimal CPP is probably patient, time, and pathology specific and related to cerebral autoregulation status. The fact that optimal CPP and autoregulation status varies between individual patients and over time makes it an attractive bedside tool to serve as a (simplified) model to investigate the use of autoregulation (PRx) status to fine tune or feedback clinical treatments in individual sedated TBI patients (optimal CPP concept) [1]. Evidence is emerging for the role of other monitors (representing (local) metabolism, oxygen supply/use, perfusion, neuronal functioning) that enable the intensivist to employ an individualized multimodality monitoring approach in TBI [2].

## Conclusion

The management of TBI is likely to become increasingly based on a more comprehensive monitoring and management approach rather than relying on absolute numbers of ICP and CPP in isolation. This will allow individual optimization of perfusion and therefore of oxygen and energy substrate delivery. We await further robust, high-quality evidence to support the benefits of using more sophisticated monitoring tools like the autoregulation-guided CPP concept during the ICU management of TBI. For the near future, more important is a greater understanding of the underlying pathophysiology.
